# Phylogeny of *Mycobacterium tuberculosis* Beijing Strains Constructed from Polymorphisms in Genes Involved in DNA Replication, Recombination and Repair

**DOI:** 10.1371/journal.pone.0016020

**Published:** 2011-01-20

**Authors:** Olga Mestre, Tao Luo, Tiago Dos Vultos, Kristin Kremer, Alan Murray, Amine Namouchi, Céline Jackson, Jean Rauzier, Pablo Bifani, Rob Warren, Voahangy Rasolofo, Jian Mei, Qian Gao, Brigitte Gicquel

**Affiliations:** 1 Unité de Génétique Mycobactérienne, Institut Pasteur, Paris, France; 2 Key Laboratory of Medical Molecular Virology, Fudan University, Shanghai, China; 3 Tuberculosis Reference Laboratory, National Institute for Public Health and the Environment, Bilthoven, The Netherlands; 4 Institute of Veterinary, Animal and Biomedical Science, Massey University, Palmerston North, New Zealand; 5 Mycobacterial Immunology, Scientific Institute of Public Health, Brussels, Belgium; 6 NRF Centre of Excellence in Biomedical Tuberculosis Research/MRC Centre for Molecular and Cellular Biology, Stellenbosch University, Cape Town, South Africa; 7 Unité de la Tuberculose et des Mycobactéries, Institut Pasteur de Madagascar, Antananarivo, Madagascar; 8 Department of Tuberculosis Control, Shanghai Municipal CDC, Shanghai, China; University of Delhi, India

## Abstract

**Background:**

The Beijing family is a successful group of *M. tuberculosis* strains, often associated with drug resistance and widely distributed throughout the world. Polymorphic genetic markers have been used to type particular *M. tuberculosis* strains. We recently identified a group of polymorphic DNA repair replication and recombination (3R) genes. It was shown that evolution of *M. tuberculosis* complex strains can be studied using 3R SNPs and a high-resolution tool for strain discrimination was developed. Here we investigated the genetic diversity and propose a phylogeny for Beijing strains by analyzing polymorphisms in 3R genes.

**Methodology/Principal Findings:**

A group of 3R genes was sequenced in a collection of Beijing strains from different geographic origins. Sequence analysis and comparison with the ones of non-Beijing strains identified several SNPs. These SNPs were used to type a larger collection of Beijing strains and allowed identification of 26 different sequence types for which a phylogeny was constructed. Phylogenetic relationships established by sequence types were in agreement with evolutionary pathways suggested by other genetic markers, such as Large Sequence Polymorphisms (LSPs). A recent Beijing genotype (Bmyc10), which included 60% of strains from distinct parts of the world, appeared to be predominant.

**Conclusions/Significance:**

We found SNPs in 3R genes associated with the Beijing family, which enabled discrimination of different groups and the proposal of a phylogeny. The Beijing family can be divided into different groups characterized by particular genetic polymorphisms that may reflect pathogenic features. These SNPs are new, potential genetic markers that may contribute to better understand the success of the Beijing family.

## Introduction

*Mycobacterium tuberculosis* is one of the most successful human pathogens, infecting nearly one third of the world's population. Despite efforts to combat the disease, tuberculosis (TB) remains a major public health problem, causing over 9 million new cases and 1.7 million deaths each year [1]. Polymorphic genetic markers have been used to discriminate and subtype *M. tuberculosis* strains to identify outbreaks. IS*6110* restriction fragment length polymorphism typing is one of the most widely used methods, however, this technique is time consuming, technically demanding and insufficiently discriminatory for isolates containing less than five copies of IS*6110*. This has led to the development of other methods based on the polymorphism of repetitive sequences, either the direct repeat (DR) region (spoligotyping) or mini satellites (variable numbers of tandem repeats (VNTR) typing) [2]. Various *M. tuberculosis* families, such as the Beijing family, have been defined using these typing techniques [3]. The Beijing family represents a global threat to TB control. It is estimated that more than a quarter of worldwide TB cases are caused by Beijing strains [3], [4]. These strains have frequently been associated with drug resistance and their emergence and wide distribution suggests they have selective advantages over other *M. tuberculosis* strains [4], [5]. Beijing strains have a characteristic spoligotype pattern [3], [5] and VNTRs have been frequently used to type these strains, exhibiting differing discriminatory abilities per VNTR locus [6], [7].

The availability of whole-genome sequences has enabled comparative genomic analysis to identify single nucleotide polymorphisms (SNPs). SNPs have been used to differentiate between clinical isolates and are preferred over the use of repeats for the construction of phylogenetic trees, because recombination events that could occur independently at the level of repetitive sequences are avoided [8]. Large numbers of SNPs have been identified and used to genotype worldwide strain collections. This supported the grouping of *M. tuberculosis* into major families and provided useful information about the evolutionary history of this monomorphic bacteria [9], [10], [11]. As an example, the phylogeny of *M. tuberculosis* was recently established by sequencing 89 genes [12]. Nevertheless, detailed phylogenies about the various *M. tuberculosis* lineages are still lacking.

We recently identified a group of highly polymorphic genes involved in DNA replication, recombination and repair (3R) in a set of geographically diverse *M. tuberculosis* strains. We showed that the evolution of *M. tuberculosis* could be studied using SNPs in 3R genes and a potential, new, high-resolution tool for strain discrimination was developed [13]. Here we investigated the genetic diversity among Beijing family strains and searched for new polymorphisms in this family by sequencing 3R genes in a collection of Beijing strains from different geographic origins in order to disclose the phylogeny of the Beijing family.

## Results and Discussion

**Figure 1:**
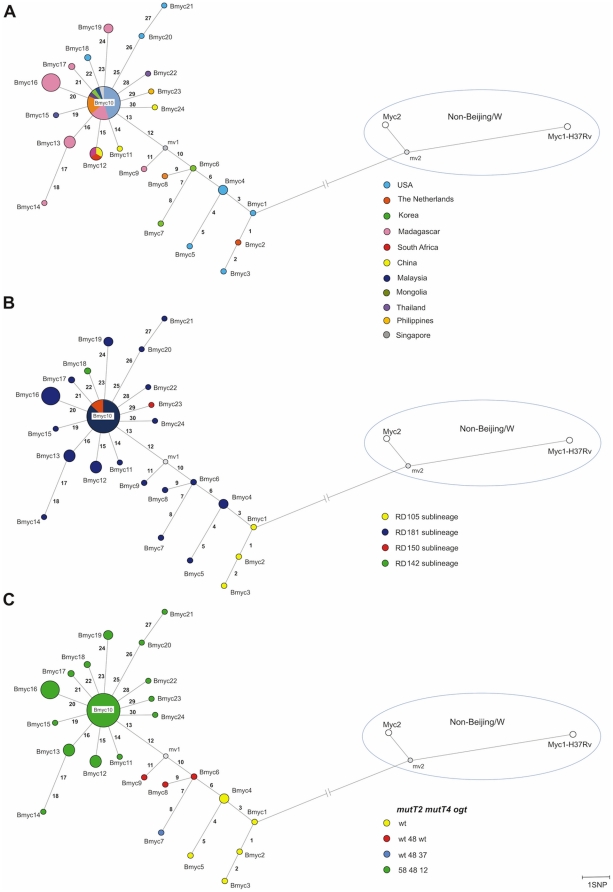
Phylogenetic network based on SNPs discovered in the collection of 58 Beijing isolates. This phylogenetic network was constructed using the median-joining algorithm with the final set of 48 SNPs characterized by sequencing 22 3R genes in 58 Beijing isolates plus one non-Beijing isolate (Myc2). Isolates are color coded according to their geographic origin (**A**), large sequence polymorphisms (LSPs) (**B**) and, variations in *mutT2 mutT4* and *ogt* genes (**C**). The reference strain *M. tuberculosis* H37Rv (Myc1) was also included. The numbers in each branch correspond to SNPs (Table 1) that enabled discrimination of sequence types. Node sizes are proportional to the number of isolates belonging to the same sequence type: Bmyc4 node (2); Bmyc12 node (3); Bmyc13 node (3); Bmyc19 (2); Bmyc16 node (7); Bmyc10 node (23). See Table S1 for details about strains belonging to each node. Mv represents a median vector created by the software and can be interpreted as possibly extant unsampled sequences or extinct ancestral sequences.

A collection of 58 clinical isolates with a Beijing spoligotype [3] was used to search for variations in 3R genes. These isolates had different geographic origins: Madagascar (19), USA (18), The Netherlands (6), South Korea (2), South Africa (2), China (3), Malaysia (1), Mongolia (2), Thailand (2), Philippines (1), Singapore (1) and Russia (1) (Table S1). These Beijing isolates included the four different sublineages defined by large sequence polymorphisms (LSPs) previously described [14] (Figure 1B). Two non-Beijing *M. tuberculosis* strains, designated Myc1, which corresponds to the laboratory strain H37Rv, and Myc2 a clinical strain that belongs to Gutacker's cluster VI [11], were also included in this study.

Of the 56 described genes encoding 3R components [13], 22 were previously demonstrated to be polymorphic among Beijing strains [13], [15]. These 22 genes (Table S2) were sequenced for each of the 58 Beijing isolates and the non-Beijing strain Myc2, resulting in approximately 1,6 Mbp of sequence data. Comparative analysis with the *M. tuberculosis* H37Rv (Myc1) genome sequence identified 48 SNPs (Table S2). Forty-one (85%) SNPs appeared to be specific for Beijing strains, as these were absent from the non-Beijing strain included in this study (Myc2) (Table S2), and also from the 86 non-Beijing *M. tuberculosis* strains included in a previous study [13]. Nineteen (46%) of these SNPs corresponded to new variations, not previously described in Beijing strains [12], [13], [15]. Thirty of the 41 Beijing specific SNPs (Table 1) enabled discrimination of 24 different sequence types for which a phylogenetic network was constructed using the Network software [16] (Figure 1A). Based on the inferred proteins, the number of non-synonymous SNPs (nsSNPs) was twice the number of synonymous SNPs (sSNPs) (Table 1). Phylogenetic relationships established by sequence types were in agreement with evolutionary pathways suggested by LSPs [14] and by SNPs in the putative DNA repair genes *mutT2*, *muT4* and *ogt* [15] (Figure 1B and 1C). However, sequencing of the 22 genes was more discriminatory than LSPs; 24 sequence types versus four sublineages defined by the LSPs.

**Table 1:** Description of SNPs that enabled discrimination of the 26 sequence types among 305 *M. tuberculosis* Beijing strains (Figures 1 and 2).

SNP number	Gene	Codon position	SNP type
1*	*ligD*	580 (CTG>TTG)	Synonymous
2	*ligD*	162 (GAT>GCT)	Non-synonymous
3*	*recR*	44 (GGT>TGT)	Non-synonymous
4	*ligD*	346 (GGC>GGT)	Synonymous
5	*uvrC*	388 (CGG>CGC)	Synonymous
6*	*mutT4*	48 (CGG>GGG)	Non-synonymous
7*	*ogt*	37 (CGC>CTC)	Non-synonymous
8	*uvrC*	166 (CAG>AAG)	Non-synonymous
9	*recX*	8 (CCG>CTG)	Non-synonymous
10*	*recX*	59 (GTT>CTT)	Non-synonymous
11	*recG*	285 (CCT>TCT)	Non-synonymous
12*	*muT2*	58 (GGA>CGA)	Non-synonymous
13*	*ogt*	12 (GGG>GGA)	Synonymous
14	*recR*	89 (GAC>GAT)	Synonymous
15*	*recF*	269 (GGG>GGT)	Synonymous
16*	*uvrD1*	462 (GGC>AGC)	Non-synonymous
17	*ligB*	77 (GTC>GCC)	Non-synonymous
18	*dnaQ*	161 (TTC>TTT)	Synonymous
19	*nth*	122 (TTG>TGG)	Non-synonymous
20*	*dnaZX*	92 (CTG>TTG)	Synonymous
21	*nth*	34 (GAG>GCG)	Non-synonymous
22	*alkA*	11 (GCG>ACG)	Non-synonymous
23*	*mutT4*	99 (TCG>TCA)	Synonymous
24*	*tagA*	129 (GCG>ACG)	Non-synonymous
25*	*recX*	153 (GGC>GAC)	Non-synonymous
26*	*radA*	276 (ATC>ACC)	Non-synonymous
27	*recD*	139 (GTA>TTA)	Non-synonymous
28	*recD*	277 (ACG>ACA)	Non-synonymous
29	*radA*	186 (GTC>GCC)	Non-synonymous
30	*tagA*	179 (GTC>GTT)	Synonymous

*Most informative SNPs observed in this study.

Next we investigated the set of 30 polymorphic SNPs (Table 1), discovered by sequence analysis of the 3R genes, in a larger collection of Beijing strains including 192 Beijing clinical isolates from China and 55 Beijing strains isolated in South Africa (Table S1). The *M. tuberculosis* Beijing strain, GC 1237, responsible for a tuberculosis epidemic in Gran Canaria, Spain [17] was also included.

**Figure 2:**
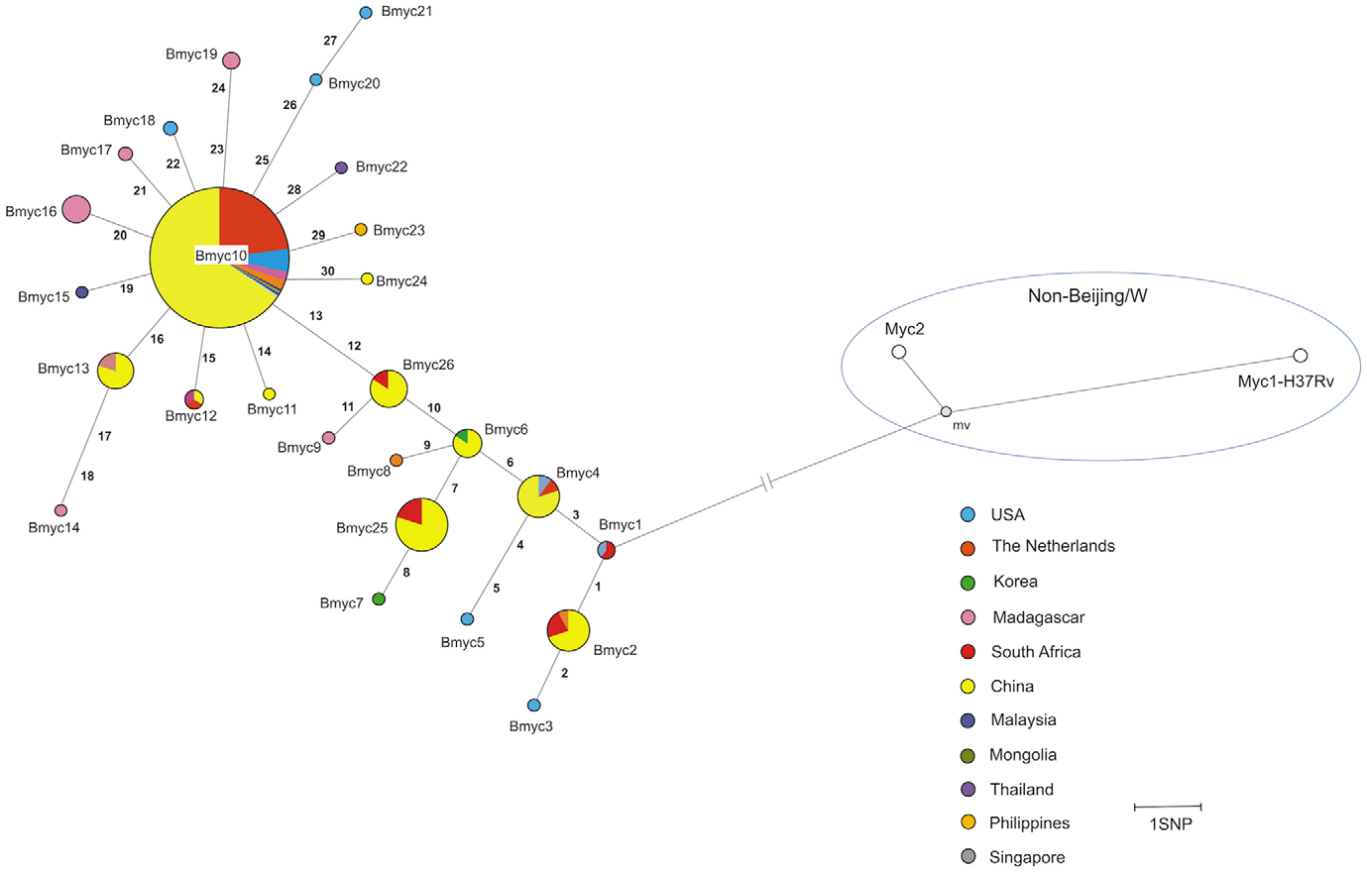
Phylogenetic network based on SNPs charaterized in the entire collection of 305 Beijing isolates. This phylogenetic network was constructed using the median-joining algorithm with the set of SNPs identified in the 3R genes analyzed on the final collection of 305 Beijing isolates. Isolates are color coded according to their geographic origin. *M. tuberculosis* strains Myc1 (H37Rv) and Myc2 are included as non-Beijing strains. The numbers in each branch correspond to SNPs (Table 1) that enabled discrimination of SNP types. Node sizes are proportional to the number of isolates belonging to the same SNP type: Bmyc1 node (2); Bmyc2 node (14); Bmyc4 node (13); Bmyc6 node (7); Bmyc25 node (28); Bmyc26 node (13); Bmyc12 node (3); Bmyc13 node (13); Bmyc16 node (7); Bmyc19 node (2); Bmyc10 node (188). See Table S1 for details about strains belonging to each node. Mv represents a median vector created by the software and can be interpreted as possibly extant unsampled sequences or extinct ancestral sequences. The relative proportion of isolates in each node, of a given geographic origin, may not reflect the population structure of the Beijing family of that geographic region.

A phylogenetic network was constructed from this larger set of isolates (Figure 2). Certain SNPs that were previously found in a single isolate, were confirmed with this larger sample. Overall, fourteen SNPs were found in more than one isolate and were therefore informative (Table 1). Two new sequence types (Bmyc25 and Bmyc26) were identified (Figure 2).

The Beijing family can be divided into different groups characterized by particular SNPs. However, a recent sequence type, represented by the Bmyc10 node, appeared to be predominant in this family (Figure 2). Sixty-two percent of the isolates belonged to this group. This sequence type was found not only in China, where the Beijing family is highly prevalent, but also in other countries, where the Beijing family is less prevalent, such as Madagascar, The Netherlands and South Africa. In a recent study, a group of Beijing strains characterized by RD181 deletion and polymorphisms in *mutT4* and *mutT2* appear to be predominant in a collection of strains isolated in Italy [18]. Strains belonging to the Bmyc10 node also had the RD181 deletion and the same SNPs in *mutT4* and *mutT2* genes (SNP6 and SNP12). This suggests that this might indeed be a prevalent group of Beijing strains which can be found in different parts of the world. The effect on enzyme characteristics of the variation in the *mutT2* gene (a characteristic of all isolates found in the R1 node, (SNP12, Figure 2)) has been investigated [19]. The results revealed significant changes in enzyme properties caused by a single amino acid substitution that leads to protein destabilization. It was suggested that this altered MutT2 enzyme may contribute to the success of strains due to an increase in nucleotide-dependent reactions. This suggests that the SNPs that we have discovered may have an effect on protein function and consequently confer advantageous phenotypes. Considering the high percentage of nsSNPs found (Table 1) it may be informative to investigate which of these variants might have a functional effect. They may confer advantageous phenotypes on certain Beijing genotypes, and play an important role in the evolution of the family. Our results showed that the Bmyc25 group might represent another predominant group of Beijing strains. This includes the Gran Canaria TB outbreak strain GC 1237 [17]. These observations suggest that several Beijing subtypes may be the result of the resurgence of tuberculosis in different regions.

When compared to other pathogens, *M. tuberculosis* complex strains are highly clonal, sharing 99% similarity at the nucleotide level [20]. In recent years, SNPs have been identified and used in order to get a more detailed insight into the evolutionary history of this organism [9], [10], [11], [12]. SNP analysis is a simple and relatively fast way to compare organisms and trace back the evolutionary history of strains, as some SNPs are highly informative. The increasing number of genome sequencing projects is making SNP analyses more and more attractive. This will provide important data, particularly relevant to understanding the genetic basis for strain differences in pathogenesis. Allelic variation in 3R genes seems to be an important mechanism in evolution and adaptation of microorganisms. Therefore, defective 3R systems could potentially increase genomic variability due to higher mutation rates. Strains with higher mutation rates (mutators) may, under certain conditions, have a selective advantage. For example, a strain may acquire mutations that induce antibiotic resistance or facilitate evasion of the host immune response [21]. The evolutionary history of a collection of 305 Beijing isolates was investigated by analyzing polymorphisms in 3R genes. We found SNPs in 3R genes associated with the Beijing family. These SNPs enabled discrimination of 26 different groups enabling a phylogenetic network to be constructed. The Beijing family can be divided into different groups presenting specific polymorphisms that may reflect pathogenic features. These new SNPs are potential genetic markers for Beijing strains that may contribute to a better understanding of the role of the Beijing family in the worldwide epidemic of tuberculosis.

## Materials and Methods

*M. tuberculosis* Beijing clinical isolates included in this study are listed in Table S1. DNA from the 58 Beijing isolates, used to search for variations in 3R genes, was provided by the Madagascar Pasteur Institute (MG), RIVM, The Netherlands (NL), Scientific Institute of Public Health, Belgium (BE) and was used to amplify the 22 3R genes with primers listed in Table 2. These fragments were sequenced by the dideoxy chain-termination method using the Big Dye Terminator v3.1 cycle sequencing Kit (Perkin Elmer Applied Biosystems, Courtaboeuf, France) according to the manufacter's instructions. Sequencing products were run on an ABI prism 3100 Genetic Analyser (Applied Biosystems). Sequencing was also performed for SNP analysis of the non-beijing strain (myc2), the Bejing isolates from South Africa (ZA) and the GC 1237 strain (DNA provided from NRF Centre of Excellence in Biomedical Tuberculosis Research/MRC Centre for Molecular and Cellular Biology, South Africa and available in our laboratory).

**Table 2:** List of oligonucleotides (5′-3′) used in this study.

Primer name	Sequence	Primer name (mismatch)	Sequence
**ligD_f**	GTCACGGCGAAATTCCACGCGATATTTGA	**ligD580_f**	CGGGCATTGGCGGAGGATCT
**ligD_r**	CCCGACCAGATCCAGCAACGACACGTC	**ligD580_wt-f**	TGCGTTAGCTAGGGTTTCGAGCAG
**ligD_2**	TCACCAGCGGCAGCAAGGGATTGCAT	**ligD580_mt-r**	TGCGTTAGCTAGGGTTTCGAGCAA
**ligD_3**	GATACACACCGAGGACCACCCGCTGGAATA	**ligD162_f**	CGACGACCTATCCGATCATCG
**ligB_f**	CCACATAGCCCCCAGGCGGTATTGGTA	**ligD162_wt-r**	GGAAGGTGACCAACCCGATAT
**ligB_r**	CGCTTGGTCGACGAGCGTGAATCTG	**ligD162_mt-r**	GGAAGGTGACCAACCCGATAG
**ligB_2**	GGCACTCTACCGGGCAAAGGGTCTCAG	**rccR44_f**	AAGCGCCCCGCCCAGGACGTG
**ligC_f**	ACCCCAGCTTCGGGAAATACATCCTGT	**rccR44_wt-r**	CGGACATCGACCGGCTGACCG
**ligC_r**	TCGCCACACAGACGACAAGTCCCAA	**rccR44_mt-r**	CGGACATCGACCGGCTGACCT
**dnaZX_f**	CGCCGAAATCACGCCGAACGTTCA	**ligD346_f**	ACCACCATCGCGCCGTACTCA
**dnaZX_r**	CGAACGAAACAACCTGCAGCTACATCACG	**ligD346_wt-r**	ACCGCCCACGAGACCAGCACG
**dnaZX_2**	AACACCTGATCTTCATATTCGCCACCA	**ligD346_mt-r**	ACCGCCCACGAGACCAGCACA
**dnaZX_3**	CTGCTGCTGGAAGTGGTTTGCG	**uvrC388_r**	GGATCCCGAAGTGGCGGTAGT
**recD_f**	GGTGTGTTCACCTGGAACCCGCCCA	**uvrC388_wt-f**	CAACAACACAAGCTGAAGCGG
**recD_r**	GTCGCCGTGCTGTTCGTGTATGCGATGT	**uvrC388_mt-f**	CAACAACACAAGCTGAAGCGC
**recD_2**	TCTCGCAAGGTGTTACGGTGTTGACTGG	**mutT4-48_r**	CGCATCAAATAATGGTGGACG
**recG_f**	CATGTGCACGACCACCATCCAGGCAC	**mutT4-48_wt-f**	CGGCAACGGCGAAGCGGTCCC
**recG_r**	CGATGATCCCAGCGTCTGATACGCGA	**mutT4-48_mt-f**	CGGCAACGGCGAAGCGGTCCG
**recG_2**	CAGCACAAAAGTGCAGAGCTGGGACATCTT	**ogt37_f**	AGCTGGGCCTGCCTGCACAAC
**recG_3**	GATGACGGCAGGGCAGAAGAAGCAAGTTC	**ogt37_wt-r**	GGTCGGGTGTCCAGTGTGTGC
**recX_f**	CCGACGTGGCTGACGAGATCGAGAAGAA	**ogt37_mt-r**	GGTCGGGTGTCCAGTGTGTGA
**recX_r**	CCGCCATCAAGTCGAGGTAAATTCGTTCA	**uvrC166_f**	CCGCTACCGCGACGACAAGTC
**ruvB_f**	GATACGGTGCTGGCCGCCAACCAT	**uvrC166_wt-r**	GCAGGCATGGACGATCGATCTG
**ruvB_r**	GGGGTCATTGCCAACGGCTCCTTTG	**uvrC166_mt-r**	GCAGGCATGGACGATCGATCTT
**uvrC_f**	CAATGCACCCGACCAACAGTGGGATAGC	**recX8_r**	GGCCGAGTTCGACATCCTCTA
**uvrC_r**	CCGGACAGCCCGGTTACCAAGACGA	**recX8_wt-f**	CTTCGCGCTCAGAAGTCGACG
**uvrC_2**	TACATCGACAAATGTTCCGCGCCGTGT	**recX8_mt-f**	CTTCGCGCTCAGAAGTCGACA
**uvrC_3**	CGGTGCACCGAAACGCAGAAGATGC	**recX59_wt-f**	GGTGTCATCCACCAGGCCAAC
**recR_f**	AAGATGGCGCAGGAACGGCTGGGT	**recX59_mt-f**	GGTGTCATCCACCAGGCCAAG
**recR_r**	GAGATCAACATTTTGCAGGCAAGGTGCG	**recX59-r**	CTCGGCCAGGGCAAGGAGAAT
**nei_f**	TCTGGTCGAGCGGGCCGACGGCAT	**recG285_r**	CGTGCGGCAGGTGCTCGATGT
**nei_r**	GGTGGCAGGCAATATCTGCCCAAGGCGG	**recG285_wt-f**	TCCCGCCGTCAGCTCAAAAGG
**nth_f**	ATGACACAAGGAGAGTAAACATGGC	**recG285_mt-f**	TCCCGCCGTCAGCTCAAAAGA
**nth_r**	AATAGTCATGCAGTTGGGCAACCA	**mutT2-58_f**	CCGGCCATAAACGTCGGAAAC
**rv2979_f**	GTTCGAAGGTCCACAGGGCCAGAACG	**mutT2-58_wt-r**	GAGGTCGGCGACCTCGAGTCC
**rv2979_r**	TCCAGTTGTATGCCTTGCGACGAGCA	**mutT2-58_mt-r**	GAGGTCGGCGACCTCGAGTCG
**tagA_f**	TGAGCTCGAGGCGCTACGCTCTCAGC	**ogt12_f**	CCGCAGGAGAAGATCGCAT
**tagA_r**	CCCCGCCATTGGATTTCCAGCCATA	**ogt12_wt-r**	GCCCGGCCAGGGTTAATAGC
**uvrD1_f**	CCCGCAAAAACTTGGCGGGAAAAGTG	**ogt12_mt-r**	GCCCGGCCAGGGTTAATAGT
**uvrD1_r**	GGACTTAGCGTCGGCAATTACACCGGTTGA	**recR89_f**	CGGACGCGATCCGTGTGACGG
**uvrD1_2**	CAACCTGAAGAACGAGTTGATCGACCC	**recR89_wt-r**	GTGCATTGTCGAGGAACCCAAAGAC
**uvrD1_3**	CGAGGGTAGCGAGATCACCTACAACGAT	**recR89_mt-r**	GTGCATTGTCGAGGAACCCAAAGAT
**dnaQ_f**	CGGGTGGTTACCACCCGGGCAGTTTAC	**recF269_f**	GGCGGAGCACGGGGCTGAACT
**dnaQ_r**	TCTCGCAAGGTGTTACGGTGTTGACTGG	**recF269_wt-r**	CGGTGCGGACCAACTAGACAAACC
**radA_f**	TAATGGTGCCGATCTCGGCCGGATT	**recF269_mt-r**	CGGTGCGGACCAACTAGACAAACA
**radA_r**	GTTGCTGCATAGCGGACATCGAGGGAGAA	**uvrD1-462_f**	TCCGCGCCGGTATTCCGTACA
**radA_2**	GAGATCTACCTCGCCGCACAGTCCGA	**uvrD1-462_wt-r**	GACGAGCGCGTCACCGAAGCC
**recF_f**	GGAGCGAGTGTCTTTCGGGTTTACGACTGC	**uvrD1-462_mt-r**	GACGAGCGCGTCACCGAAGCT
**recF_r**	CGCCCTCGACCGGCGTCTTGTCC	**ligB77_r**	TGTCGGCGAGACATGCCAAGCT
**mutT2_f**	CTGCCAGCCGTTGAGGTCGT	**ligB77_wt-f**	GGGTGGCGTCGACACCGGTGA
**mutT2_f**	CGGGCATGCAAACCCAAGTTA	**ligB77_mt-f**	GGGTGGCGTCGACACCGGTGG
**mutT4_f**	TCGAAGGTGGGCAAATCGTG	**dnaQ161_f**	GGACCAGCGGGCGGCCCTGGA
**mutT4_r**	TGGGGTTCGCTGGAAGTGG	**dnaQ161_wt-r**	CAACGGCCGCACGATGCATTC
**ogt_f**	CAGCGCTCGCTGGCGCC	**dnaQ161_mt-r**	CAACGGCCGCACGATGCATTT
**ogt_r**	GACTCAGCCGCTCGCGA	**nth122_f**	CCCGCCGTCCGTGAAAGATCA
**alkA_f**	AGCCGCGTAGGTAACCT	**nth122_wt-r**	GCCGGCCACCATGGACAAGTT
**alkA_r**	TGCTCGAGCATCCGCAG	**nth122_mt-r**	GCCGGCCACCATGGACAAGTG
**alkA_2**	CGCATGCAGACCGCCCG	**nth34_f**	TCACCGCCAAACCGCTCAA
**alkA_3**	CACTGCACGTTGCCGAC	**nth34_wt-r**	ATTTCCGCACGTATACTGAGA
**alkA_4**	GCTGACGATGCCGTTGCC	**nth_34_mt-r**	ATTTCCGCACGTATACTGAGC
		**dnaZX92_f**	GCGAGCAACGCCCGCATAGT
		**dnaZX92_wt-r**	GCAGCATCGACGTGGTAGAGC
		**dnaZX92_mt-r**	GCAGCATCGACGTGGTAGAGT
		**alkA11_f**	ATCGCCCGCGCCACGACGTCA
		**alkA11_wt-r**	ACTTCGAACGCTGCTACCGGG
		**alkA11_mt-r**	ACTTCGAACGCTGCTACCGGA
		**mutT4-99_f**	GGCGGCGCGCTGCGGCTACAG
		**mutT4-99_wt-r**	CAACTCGATGTGCCCCTTGGGTAGC
		**mutT4-99_mt-r**	CAACTCGATGTGCCCCTTGGGTAGT
		**recX153_f**	GCGGGCGAACGCAGCAAAGAG
		**recX153_wt-r**	CCTCGCACGCCAAGGTCTGGC
		**recX153_mt-r**	CCTCGCACGCCAAGGTCTGGT
		**recD277_r**	CGGGCCTGGCACCGGGAAGAC
		**recD277_wt-f**	TCGGCCAGCCGGGCCATCAGC
		**recD277_mt-f**	TCGGCCAGCCGGGCCATCAGT
		**radA186_f**	TCCGGACGGCGCGCGCTCTAT
		**radA186_wt-r**	CCTGCGTGACCCCGCCGGTGA
		**radA186_mt-r**	CCTGCGTGACCCCGCCGGTGG
		**tagA179_f**	GTGCGCAACCGCGCCAAGATT
		**tagA179_wt-r**	CCAGCATGCTTGGATATGGTCGTCG
		**tagA179_mt-r**	CCAGCATGCTTGGATATGGTCGTCA

The name of the target gene and position of the oligonucleotide is followed by the oligonucleotide sequence. (f) for forward and (r) for reverse oligonucleotides used for amplification and sequencing reactions. Oligonucleotides whose name finishes in number were used for sequencing reactions. (wt) for wild-type and (mt) for mutant oligonucleotides used for detection of SNPs by mismatched PCR (see materials and methods).

Sequences were analysed using the software Genalys obtained at http://software.cng.fr/. The genome sequences of *M.tuberculosis* H37Rv were obtained from the Institut Pasteur at http://genolist.pasteur.fr and used for detection of SNPs.

A mismatched PCR method, using one wild-type primer and one containing the SNP which matched/mismatched the template DNA at the 3′-end of the primer (Table 2), was used to detect SNPs in the Beijing isolates from China (CN).

SNPs were concatenated resulting in one character string (nucleotide sequence) for each clinical isolate analyzed. A FASTA file was created to run in the Network software [16] to build a phylogeny based on the median-joining method. This software assumes that there is no recombination between genomes.

## Supporting Information

Table S1 **Description of** ***M. tuberculosis*** **Beijing strains belonging to each node found in** **Fig. 1** **and** **2** **, and respective country of isolation.**
(DOC)

Table S2 **Full list of 48 SNPs identified in this study.** The first line indicates the gene and the second line indicates the position on that gene where polymorphisms were identified in relation to *M. tuberculosis* H37Rv strain (bottom). Polymorphisms that characterize and allowed discrimination of the 26 sequence types (Figure 2 and Table 1) are marked in red.
(XLS)
