# Supplementary material for: Phylogeny of Mycobacterium tuberculosis Beijing Strains Constructed from Polymorphisms in Genes Involved in DNA Replication, Recombination and Repair
Source: PLoS One. 2011 Jan 20;6(1):e16020. doi: 10.1371/journal.pone.0016020 (PMC3024326; doi:10.1371/journal.pone.0016020)
Supplement: Table S1 — Description of M. tuberculosis Beijing strains belonging to each node found in Fig. 1 and 2 , and respective country of isolation. (DOC) [file pone.0016020.s001.doc]

**Table S1.** Description of *M. tuberculosis* Beijing strains belonging to each node found in Fig. 1 and 2, and respective country of isolation.

| **Node** | **Strain** | **Origin of isolation** |
| --- | --- | --- |
| **Bmyc1** | NL25 | USA |
|  | ZA40 | South Africa |
| **Bmyc2** | NL20 | The Netherlands |
|  | CN1 | China |
|  | CN2 | China |
|  | CN3 | China |
|  | CN4 | China |
|  | CN5 | China |
|  | CN6 | China |
|  | CN7 | China |
|  | CN8 | China |
|  | CN9 | China |
|  | ZA13 | South Africa |
|  | ZA42 | South Africa |
|  | ZA43 | South Africa |
|  | ZA51 | South Africa |
| **Bmyc3** | NL34 | USA |
| **Bmyc4** | NL17 | USA |
|  | BE26 | USA |
|  | CN10 | China |
|  | CN11 | China |
|  | CN12 | China |
|  | CN13 | China |
|  | CN14 | China |
|  | CN15 | China |
|  | CN16 | China |
|  | CN17 | China |
|  | CN18 | China |
|  | ZA45 | South Africa |
|  | ZA52 | South Africa |
| **Bmyc5** | NL33 | USA |
| **Bmyc6** | NL19 | South Korea |
|  | CN20 | China |
|  | CN21 | China |
|  | CN22 | China |
|  | CN23 | China |
|  | CN24 | China |
|  | CN25 | China |
| **Bmyc7** | BE3 | South Korea |
| **Bmyc8** | NL21 | The Netherlands |
| **Bmyc9** | MG9 | Madagascar |
| **Bmyc10** | NL1 | Mongolia |
|  | NL2 | South Africa |
|  | NL3 | Malaysia |
|  | NL7 | Thailand |
|  | NL18 | The Netherlands |
|  | NL23 | The Netherlands |
|  | NL24 | The Netherlands |
|  | NL31 | The Netherlands |
|  | BE1 | USA |
|  | BE5 | Singapore |
|  | BE8 | USA |
|  | BE10 | USA |
|  | BE12 | USA |
|  | BE14 | USA |
|  | BE17 | USA |
|  | BE19 | USA |
|  | BE20 | USA |
|  | BE23 | USA |
|  | BE27 | USA |
|  | MG3 | Madagascar |
|  | MG10 | Madagascar |
|  | MG11 | Madagascar |
|  | MG21 | Madagascar |
|  | CN60 | China |
|  | CN61 | China |
|  | CN62 | China |
|  | CN63 | China |
|  | CN64 | China |
|  | CN65 | China |
|  | CN66 | China |
|  | CN67 | China |
|  | CN68 | China |
|  | CN69 | China |
|  | CN70 | China |
|  | CN71 | China |
|  | CN72 | China |
|  | CN73 | China |
|  | CN74 | China |
|  | CN75 | China |
|  | CN76 | China |
|  | CN77 | China |
|  | CN78 | China |
|  | CN79 | China |
|  | CN80 | China |
|  | CN81 | China |
|  | CN82 | China |
|  | CN83 | China |
|  | CN84 | China |
|  | CN85 | China |
|  | CN86 | China |
|  | CN87 | China |
|  | CN88 | China |
|  | CN89 | China |
|  | CN90 | China |
|  | CN91 | China |
|  | CN92 | China |
|  | CN93 | China |
|  | CN94 | China |
|  | CN95 | China |
|  | CN96 | China |
|  | CN97 | China |
|  | CN98 | China |
|  | CN99 | China |
|  | CN100 | China |
|  | CN101 | China |
|  | CN102 | China |
|  | CN103 | China |
|  | CN104 | China |
|  | CN105 | China |
|  | CN106 | China |
|  | CN107 | China |
|  | CN108 | China |
|  | CN109 | China |
|  | CN110 | China |
|  | CN111 | China |
|  | CN112 | China |
|  | CN113 | China |
|  | CN114 | China |
|  | CN115 | China |
|  | CN116 | China |
|  | CN117 | China |
|  | CN118 | China |
|  | CN119 | China |
|  | CN120 | China |
|  | CN121 | China |
|  | CN122 | China |
|  | CN123 | China |
|  | CN124 | China |
|  | CN125 | China |
|  | CN126 | China |
|  | CN127 | China |
|  | CN128 | China |
|  | CN129 | China |
|  | CN130 | China |
|  | CN131 | China |
|  | CN132 | China |
|  | CN133 | China |
|  | CN134 | China |
|  | CN135 | China |
|  | CN136 | China |
|  | CN137 | China |
|  | CN138 | China |
|  | CN139 | China |
|  | CN140 | China |
|  | CN141 | China |
|  | CN142 | China |
|  | CN143 | China |
|  | CN144 | China |
|  | CN145 | China |
|  | CN146 | China |
|  | CN147 | China |
|  | CN148 | China |
|  | CN149 | China |
|  | CN150 | China |
|  | CN151 | China |
|  | CN152 | China |
|  | CN153 | China |
|  | CN154 | China |
|  | CN155 | China |
|  | CN156 | China |
|  | CN157 | China |
|  | CN158 | China |
|  | CN159 | China |
|  | CN160 | China |
|  | CN161 | China |
|  | CN162 | China |
|  | CN163 | China |
|  | CN164 | China |
|  | CN165 | China |
|  | CN166 | China |
|  | CN167 | China |
|  | CN168 | China |
|  | CN169 | China |
|  | CN170 | China |
|  | CN171 | China |
|  | CN172 | China |
|  | CN173 | China |
|  | CN174 | China |
|  | CN175 | China |
|  | CN176 | China |
|  | CN177 | China |
|  | CN178 | China |
|  | CN179 | China |
|  | CN180 | China |
|  | CN181 | China |
|  | CN182 | China |
|  | ZA1 | South Africa |
|  | ZA2 | South Africa |
|  | ZA3 | South Africa |
|  | ZA4 | South Africa |
|  | ZA5 | South Africa |
|  | ZA6 | South Africa |
|  | ZA7 | South Africa |
|  | ZA8 | South Africa |
|  | ZA10 | South Africa |
|  | ZA11 | South Africa |
|  | ZA12 | South Africa |
|  | ZA15 | South Africa |
|  | ZA16 | South Africa |
|  | ZA17 | South Africa |
|  | ZA18 | South Africa |
|  | ZA20 | South Africa |
|  | ZA21 | South Africa |
|  | ZA22 | South Africa |
|  | ZA23 | South Africa |
|  | ZA24 | South Africa |
|  | ZA25 | South Africa |
|  | ZA26 | South Africa |
|  | ZA28 | South Africa |
|  | ZA29 | South Africa |
|  | ZA31 | South Africa |
|  | ZA32 | South Africa |
|  | ZA33 | South Africa |
|  | ZA34 | South Africa |
|  | ZA36 | South Africa |
|  | ZA38 | South Africa |
|  | ZA41 | South Africa |
|  | ZA44 | South Africa |
|  | ZA46 | South Africa |
|  | ZA47 | South Africa |
|  | ZA48 | South Africa |
|  | ZA49 | South Africa |
|  | ZA50 | South Africa |
|  | ZA53 | South Africa |
|  | ZA54 | South Africa |
|  | ZA55 | South Africa |
|  | ZA56 | South Africa |
|  | ZA57 | South Africa |
| **Bmyc11** | NL4 | China |
| **Bmyc12** | NL28 | South Africa |
|  | BE6 | Russia |
|  | BE9 | China |
| **Bmyc13** | MG8 | Madagascar |
|  | MG12 | Madagascar |
|  | CN183 | China |
|  | CN184 | China |
|  | CN185 | China |
|  | CN186 | China |
|  | CN187 | China |
|  | CN188 | China |
|  | CN189 | China |
|  | CN190 | China |
|  | CN191 | China |
|  | CN192 | China |
| **Bmyc14** | MG7 | Madagascar |
| **Bmyc15** | NL6 | Malaysia |
| **Bmyc16** | MG13 | Madagascar |
|  | MG14 | Madagascar |
|  | MG15 | Madagascar |
|  | MG16 | Madagascar |
|  | MG17 | Madagascar |
|  | MG19 | Madagascar |
|  | MG20 | Madagascar |
| **Bmyc17** | MG6 | Madagascar |
| **Bmyc18** | BE21 | USA |
| **Bmyc19** | MG4 | Madagascar |
|  | MG5 | Madagascar |
| **Bmyc20** | BE13 | USA |
| **Bmyc21** | BE16 | USA |
| **Bmyc22** | NL5 | Thailand |
| **Bmyc23** | BE15 | Philippines |
| **Bmyc24** | BE7 | China |
| **Bmyc25** | GC1237 | Spain |
|  | ZA9 | South Africa |
|  | ZA14 | South Africa |
|  | ZA19 | South Africa |
|  | ZA35 | South Africa |
|  | CN26 | China |
|  | CN27 | China |
|  | CN28 | China |
|  | CN29 | China |
|  | CN30 | China |
|  | CN31 | China |
|  | CN32 | China |
|  | CN33 | China |
|  | CN34 | China |
|  | CN35 | China |
|  | CN36 | China |
|  | CN37 | China |
|  | CN38 | China |
|  | CN39 | China |
|  | CN40 | China |
|  | CN41 | China |
|  | CN42 | China |
|  | CN43 | China |
|  | CN44 | China |
|  | CN45 | China |
|  | CN46 | China |
|  | CN47 | China |
|  | CN48 | China |
| **Bmyc26** | CN49 | China |
|  | CN50 | China |
|  | CN51 | China |
|  | CN52 | China |
|  | CN53 | China |
|  | CN54 | China |
|  | CN55 | China |
|  | CN56 | China |
|  | CN57 | China |
|  | CN58 | China |
|  | CN59 | China |
|  | ZA30 | South Africa |
|  | ZA58 | South Africa |
